# Aminolevulinate inhibition of human coproporphyrinogen oxidase clarifies coproporphyrin III accumulation in porphyrias

**DOI:** 10.1042/BSR20254015

**Published:** 2026-02-24

**Authors:** Andreas Schedlbauer, Sarah Kratzwald, Margarita Gómez-Galán, Jon Gil-Martínez, Itxaso San Juan, Tania Pereira-Ortuzar, Fernando Lopitz-Otsoa, David Fernandez-Ramos, José M. Mato, Oscar Millet

**Affiliations:** 1 Precision Medicine and Metabolism Laboratory, CIC bioGUNE, Basque Research and Technology Alliance, Parque Tecnológico de Bizkaia, Ed. 800, 48160 Derio, Spain; 2 ATLAS Molecular Pharma, Parque Tecnológico de Bizkaia, Ed. 800, 48160 Derio, Spain; 3 CIBERehd, Madrid, Spain

**Keywords:** ALAD porphyria, aminolevulinate, coproporphyrin, enzyme inhibition, lead intoxication

## Abstract

Porphyrias are inherited or acquired disorders of heme biosynthesis characterized by heme deficiency and accumulation of toxic intermediates. In δ-aminolevulinic acid dehydratase deficiency porphyria (ALADP), patients consistently present elevated urinary δ-aminolevulinic acid (δ-ALA) and coproporphyrin III (COPRO III), yet the mechanistic basis of COPRO III accumulation remains unclear. This metabolic disturbance is also observed in the porphyria-like associated crises in hereditary tyrosinemia type I (HT1). Here, we investigated the effects of δ-ALA, COPRO III, and lead (Pb^2+^) on human coproporphyrinogen oxidase (CPOX), a key mitochondrial enzyme in heme biosynthesis. Using purified recombinant CPOX, we show that COPRO III binds with high affinity (*K*_D_ ≈ 2.1 μM) and acts as a competitive inhibitor, while δ-ALA inhibits CPOX at millimolar concentrations through a non-competitive, likely covalent, mechanism. *In vivo*, δ-ALA accumulation in an HT1 mouse model led to hepatic COPRO III buildup, consistent with our *in vitro* findings and supporting a synergistic inhibition model in which δ-ALA promotes secondary COPRO III accumulation that further impairs CPOX. Additionally, Pb^2+^ was found to inactivate CPOX, likely through oxidative damage, providing a molecular explanation for enzyme dysfunction in porphyrin abnormalities in response to lead intoxication. Together, these results identify multiple metabolite- and toxin-dependent mechanisms that converge on CPOX inhibition, offering new insights into the pathophysiology of ALADP, among other porphyrias, lead intoxication, and HT1.

## Introduction

Porphyrias are a group of inherited metabolic disorders resulting from dysfunction in the heme biosynthetic pathway [1]. There are eight recognized types, broadly classified into acute hepatic porphyrias, which primarily affect the nervous system, and chronic cutaneous porphyrias, which manifest predominantly with skin symptoms [2]. Genetically, each porphyria is typically associated with a mutation in a single gene encoding one of the enzymes in the heme biosynthesis pathway, with a generally one-to-one correspondence and few exceptions [3]. Clinically, symptoms vary widely depending on the specific form of the disease [4]. Acute porphyrias are often characterized by severe abdominal pain, while chronic forms commonly manifest as cutaneous lesions and photosensitivity. Hepatic involvement is frequently observed across different subtypes [5]. At the molecular level, porphyrias are characterized by heme deficiency and the accumulation of biosynthetic intermediates, notably type III isomers, and/or by-products, such as type I isomers [6].

Doss porphyria, also known as δ-aminolevulinic acid dehydratase deficiency porphyria (ALADP), is a rare autosomal recessive form of porphyria caused by a deficiency in δ-aminolevulinic acid dehydratase (also known as porphobilinogen synthase, EC4.2.1.24, ALAD) [7]. ALAD catalyzes the asymmetric condensation of two molecules of δ-aminolevulinic acid (δ-ALA) to form porphobilinogen, a key precursor in the heme biosynthetic pathway. A defect in ALAD leads to the accumulation of the toxic precursor δ-ALA [8].

Intriguingly, patients with ALADP consistently exhibit elevated levels of coproporphyrin III (COPRO III) in urine and, to a lesser extent, in feces [9]. Intriguingly, a similar interplay between δ-ALA and COPRO III accumulation can be observed, although to a lesser extent, in other porphyrias, including variegate porphyria and harderoporphyria. In addition, studies in normal subjects have shown that administration of large doses of δ-ALA leads to coproporphyrinuria [10,11]. Finally, patients undergoing hereditary tyrosinemia type I (HT1) also present acute porphyria-like crises that include the accumulation of δ-ALA and COPRO III as well [12]. The accumulation of δ-ALA in HT1 is caused by the abnormal generation of succinyl acetone, a potent inhibitor of ALAD [13], but the accumulation of COPRO III is largely unexpected. Coproporphyrinogen III (COPROgen III), the enzymatic substrate of coproporphyrinogen oxidase (CPOX), is converted by CPOX to protoporphyrinogen IX, whereas COPRO III represents the oxidized form of COPROgen III and is therefore a non-enzymatic by-product rather than a physiological reaction product. While COPRO III accumulation has been linked to various non-porphyric conditions [14], its persistent elevation in ALADP (and in other porphyrias and in HT1) appears to be independent of disease stage [15]. COPRO III accumulation may result from the inhibition of CPOX by δ-ALA, but the physiological mechanism underlying this phenomenon remains unclear.

In addition to genetic inheritance, ALAD deficiency can also occur through environmental exposure to lead, resulting in acquired toxicity [16,17]. Human studies have shown that lead poisoning results in increased urinary excretion of ALA and coproporphyrin. Lead disrupts porphyrin metabolism by affecting the normal enzymatic activity of up to three enzymes in the heme pathway: ALAD, CPOX, and ferrochelatase [18,19]. CPOX is a dimeric protein with a tertiary structure consisting of a flat seven-stranded β-sheet sandwiched by α-helices [20], and the flat surface is supposed to serve as the docking site for the substrate. Some studies suggest that lead may inhibit CPOX [15,21]. However, these conclusions are based on assays in whole cells rather than purified enzymes, limiting the specificity of the findings.

In the present study, we aimed to evaluate the effect of δ-ALA, Pb^2^^+^, and related by-products on the enzymatic kinetics of human mitochondrial oxygen-dependent CPOX. Our results demonstrate that Pb^2+^ is a powerful inhibitor of CPOX and may explain its inhibition after lead intoxication. It elevated δ-ALA and COPRO III levels, also inhibited CPOX activity *in vitro*, supporting a model in which δ-ALA is the primary cause of enzymatic inhibition in ALADP and other porphyrias. To validate the clinical relevance of these findings, we examined a mouse model of HT1, which similarly exhibits hepatic accumulation of COPRO III in conjunction with elevated intracellular δ-ALA levels.

## Methods

### Reagents and stocks

The precursor compound COPRO III (Cat. No. C654-3, MW = 727.64 g/mol) for the preparation of the enzyme substrate and the standard compound protoporphyrin IX (Cat. No. P562-9, MW = 562.66 g/mol) were commercially purchased from Frontier Scientific. One vial (10 mg) of COPRO III was dissolved in 2 ml of argon-flushed methanol to obtain a final stock concentration of 6.9 mM. The stock solution for the reference protoporphyrin IX was prepared by dissolving 2 mg of protoporphyrin IX in 0.5 ml of 100 mM KOH by vigorous vortexing for approximately one minute before adding 0.5 ml of ethanol yielding a concentration of about 3.5 mM. The concentration of the COPRO III solution was assessed spectrophotometrically in 100 mM HCl, monitoring the 548 nm absorbance band using a molar extinction coefficient ε of 16,800 M^−1^cm^−1^, whereas the concentration of the stock solution of protoporphyrin IX was determined in 2.6 M HCl at 408 nm with a molar extinction coefficient ε of 29,700 M^−1^cm^−1^. Both solutions were stored protected from light at −20°C.

### Production and purification of human coproporphyrinogen-III oxidase

The wild-type enzyme construct, encompassing residue T111 to the C-terminal residue R454 of human CPOX (UniProt P36551), was fused via a Factor Xa cleavage site to an N-terminal 10× His tag and cloned into the pET16b vector. The gene sequences for the R183K-, R262K-, R332K-, and R413K-mutants of CPOX were cloned into pet28b vector, linked via a TEV cleavage site to an N-terminal 6× His tag. All these plasmids for protein expressions were purchased from Genescript™ and used for transformation of BL21-DE3 (Novagen) cells. To reduce the effects of protein toxicity on bacterial cell growth in the lac operon-based system prior to induction, the LB media was supplemented with 0.2% (w/v) glucose. This adjustment, coupled with the reduced metabolic rate when expression was done in minimal M9 media for uniform ^15^N isotope enrichment, led to a significant gain in the yield of soluble protein. Protein production was induced when the cultures reached an OD_600_ of 0.8, with CPOX enzymes expressed under the control of the T7 promoter at 17°C for 12–18 h, while monitoring the growth rate. The M9 minimal growth media (24 mM Na_2_HPO_4_, 11 mM KH_2_PO_4_, 4.3 mM NaCl, 2 mM MgSO_4_, 0.1 mM CaCl_2_, 200 mg/l thiamine hydrochloride, and 4 g/l glucose) was provided with trace metal mix composition according to F. Studier, along with 1 g/l ^15^NH_4_Cl (≥98 atom% ^15^N, Sigma–Aldrich) as the only nitrogen source.

For protein purification, the cell pellet was thawed and resuspended in lysis buffer (25 mM Tris–HCl, pH 8.0, 150 mM NaCl, 12.5 mM imidazole, 0.2 mg/ml lysozyme, 0.5% Triton X-100, 300 μM TCEP, and 10% glycerol) at a (w/v) ratio of approximately ∼6:1 (v/w) buffer-to-pellet ratio. After an additional freeze-thaw cycle, the sample was incubated on ice for 30 min before being subjected to high-speed centrifugation at 118,700***g*** for 2 h at 4°C. The supernatant was filtered through a 0.2-μm membrane (pre-washed with lysis buffer) and loaded onto a 3 ml HisTrap resin column equilibrated with loading buffer A (50 mM Tris, pH 8.0, 350 mM NaCl, 25 mM imidazole, 300 μM TCEP, and 10% glycerol). The bound CPOX enzyme was washed extensively with loading buffer (approximately 30 column volumes) before eluting with buffer B (same as buffer A but containing 250 mM imidazole). To remove the N-terminal His tag, the concentrated supernatant (using a Vivaspin device with a 10 kDa cutoff) was transferred into gel filtration buffer (50 mM phosphate, pH 7.0, 150 mM NaCl, 10 mM DTT, and 10% glycerol) using a Sephadex™ G-25 column. The sample was incubated overnight with Factor Xa or TEV protease at 4°C before loading onto an SD200 (16/60) gel filtration column equilibrated with gel filtration buffer (same as assay buffer in the next subsection). The protein eluted around 86 ml, and its purity was confirmed by gradient SDS–PAGE (4.5%–20%) (Supplementary Figure S1). Protein concentration was determined using a NanoDrop™ UV-Vis spectrophotometer with a molar extinction coefficient ε of 51,910 M^−1^cm^−1^ and confirmed by the bicinchoninic acid (BCA) protein assay (Thermo Scientific).

### Assaying human CPOX protein activity

The substrate was transferred into assay buffer and stored under argon at −80°C until use. Solvents used were extensively deoxygenated by argon flush. All working steps for the substrate generation and for the enzymatic assay were conducted in an anaerobic chamber under argon atmosphere and a dark-room red light. An amount of 5 mg Pd/C (10% Pd by weight on activated carbon), 200 μl of 0.1 M NH_4_OH, 1400 μl of methanol, and 400 μl of 6.87 mM COPRO III were evacuated and flushed with argon twice before exposing the mixture to hydrogen gas from a fully inflated gas bag. The mixture was vigorously stirred for approximately 40 min.

After removal of the Pd/C catalyst by filtration through a 0.2-μm membrane, the solvent was removed by argon flushing at 40°C. The resulting dry Pd/C-reduced COPROgen III was dissolved in deoxygenated assay buffer, flash-frozen in liquid nitrogen, and stored at −80°C until use.

The enzymatic assay was performed in a total volume of 150 μL containing 20 μM CPOX enzyme and up to 500 μM COPROgen III dissolved in deoxygenated assay buffer (50 mM phosphate, pH 7.0; 150 mM NaCl; 10 mM DTT; and 10% glycerol). The reaction mixture was incubated at 37°C for 1 h and stopped by addition of 200 μl of 20% (w/v) trichloroacetic acid in 50% (v/v) dimethyl sulfoxide.

The product of the oxidase reaction was then exposed to ambient laboratory conditions and light for approximately one hour before centrifugation at 14,000***g*** for 30 min. The supernatant was collected for HPLC analysis. The formed protoporphyrin IX was separated from the reoxidized substrate COPRO III using a LiChrospher 100 RP-18 column with a gradient elution consisting of 90% (v/v) methanol in 1 M ammonium acetate buffer (pH 5.16) and methanol. Detailed chromatographic conditions and detection parameters are provided in Supplementary Table S1. Baseline correction and signal quantification were performed using custom Python scripts employing the *hplc-py* package.

### Circular dichroism experiments

Thermal stability analysis of the protein and estimation of the associated thermodynamic parameters upon COPRO III binding were performed using circular dichroism (CD) spectroscopy. The experiments were conducted using a Jasco-815 CD spectrometer equipped with a photomultiplier tube detector and CDF 426s Peltier temperature control system. Initially, the ellipticity (Θ) as a function of wavelength was recorded to identify the negative maxima most sensitive to changes in protein secondary structure. The CD signal at this wavelength was then monitored across increasing temperatures to determine the melting temperature, *T*_m_, corresponding to the secondary structure unfolding. The temperature dependence of the CD signal, Θ(T), during the unfolding process was fitted to a sigmoidal curve (using a custom Python script), under the assumption of a two-state transition as previously described [22]. The change of heat capacity ΔCp was approximated by (0.19·(93·aa-907)-251)·4.18·exp^−3^ in J/(mol K), with aa equaling the number of amino acids in the primary sequence of the protein. A summary of the parameters used for the CD measurements is provided in Supplementary Table S2.

The fitting of the *T*_m_ dependence with the COPRO III concentration employed the following expression (eqn 1):

(1) $$\begin{array}{l} \Delta T_{m}=\Delta T_{m}^{\max}\cdot ([COPROIII]+P_{T}+K_{D})-\\ \sqrt{{\left([COPROIII]+P_{T}+K_{D}\right)}^{2}-(4\cdot [COPROIII])\cdot P_{T}/2\cdot P_{T}} \end{array},$$

where *P*_T_ is the total protein concentration and *K*_D_ is the dissociation constant.

### NMR spectroscopy

Quantitative ^1^D NMR spectra were acquired using *noesygppr1d* sequence for enhanced sensitivity and resolution using relaxation delay of 10 s, a spectral width of 16 ppm, 16 k data points and 32 scans per increment. Proton chemical shifts were directly referenced to added DSS (2,2-dimethyl-2- silapentane-5-sulphonic acid). All NMR data were processed with NMRpipe [23] and analyzed using CCPNMR [24].

### Modeling coproporphyrinogen III- and coproporphyrin III- bound CPOX

The crystal structure of the human CPOX enzyme (pdbID = 2AEX) processed using AutoDockTools protonated to simulate physiological pH conditions served as a receptor template. The topologies of the ligands (COPRO III, COPROgen III, δ-ALA and citrate) were generated using RDKIT tools and Meeko, and their ionization states were enumerated using Dimorphite-DL script (version 1.2.4). The docking box (18 × 18 × 16 Å) was centered on the active site of CPOX, identified from the crystal structure and literature references. The docking simulations were performed for 100 iterations with a maximum number of poses to generate 10 conformations of the ligand utilizing the Lamarckian genetic algorithm. The best binding poses obtained were subjected to further refinement by molecular dynamics simulations using openMM software [25] and MDAnalysis library [26].

### Animal experiments

All procedures involving animals were in accordance with the Spanish Guide for the Care and Use of Laboratory Animals and with International Animal Care and Use Committee Standards, and they were approved by the CIC bioGUNE ethical review committee (Permit Number P-CBG-CBBA-0721 for animal breeding and P-CBG-CBBA-2321 and P-CBG-CBBA-0424 for animal characterization and interventions). CIC bioGUNE animal facility holds an AAALAC accreditation. Due to the recessive nature of the disease, wild-type and heterozygous animals were considered in the same group. Mice in the nitisinone (NTBC)-treated group were put in separate cages with 7.5 mg/l NTBC in their drinking water *ad libitum* since pregnancy.

### Sample extraction from mice

Mice urine was collected by gently immobilizing each mouse to obtain fresh urine or obtained directly from the bladder after euthanasia (see expanded experimental section). While anesthetized, mice were killed by cervical dislocation, and organs were collected, flash-frozen in liquid nitrogen, and stored at −80°C.

### Porphyrin measurement

Frozen liver samples (50 mg) were lysed in 200 μl of 6 M HCl, incubated for 30 min at 37°C, and centrifuged 10 min at 10,000 rcf. Supernatants were transferred to a Spin-X 0.22 μm pore size cellulose acetate filter tube (Corning) and centrifuged for 10 min at 3500 rcf. Filtered samples were separated by HPLC as previously described [27].

## Results

### Substrate synthesis and *in vitro* CPOX enzymatic assay

**Figure 1:**
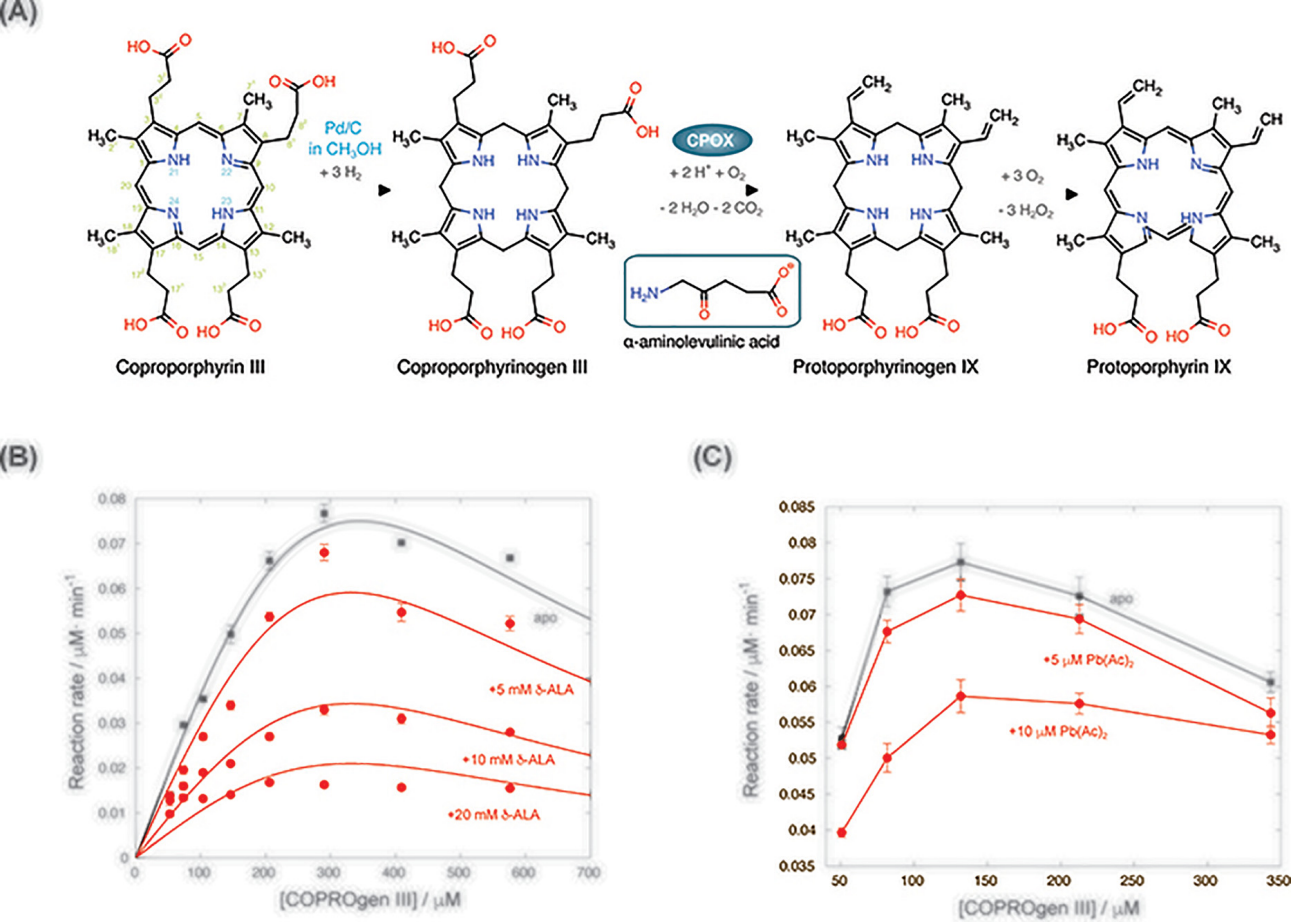
CPOX enzymatic reaction (**A**) Scheme representing the enzymatic reaction assay. The δ-ALA structure is shown in the square inset. (**B**) apo CPOX and the effect of δ-ALA (red) on CPOX enzyme kinetics. δ-ALA concentrations are indicated in the legend. Solid lines represent the best fits to the enzymatic models. (**C**) apo CPOX and the effect of lead (red) on CPOX enzyme kinetics. Pb(Ac)_2_ concentrations are indicated in the legend.

To evaluate the enzymatic activity of CPOX, its substrate COPROgen III (highly prone to oxidation) was first prepared by catalytic reduction of commercially available COPRO III (see Figure 1A). The hydrogenation of COPRO III to COPROgen III was carried out using a palladium on carbon (Pd/C) catalyst (10% by weight on activated carbon), which was readily removed by filtration due to its particle size (∼30 μm). The filtrate was then evaporated to yield dry COPROgen III, which was subsequently dissolved in a buffer suitable for the enzymatic assay.

CPOX catalyzes the oxidation of COPROgen III to protoporphyrinogen IX using molecular oxygen. However, the *in vitro* assay is challenging because direct chemical oxidation may also occur (Figure 1A). Precise oxygen control during the metal- and cofactor-independent decarboxylation catalyzed by CPOX proved essential to minimize chemical reoxidation in a reproducible manner. This was achieved by maintaining an optimal and constant oxygen partial pressure in all buffers (see the ‘Methods’ section). The purity of the prepared substrate and the absence of side products or contaminants were confirmed by HPLC analysis (Supplementary Figure S2).

Under these experimental conditions, we determined the initial rates of CPOX activity at increasing COPROgen III concentrations (Figure 1B, black squares). The results showed that the conversion rate decreased at high substrate concentrations. We hypothesize that part of the substrate is converted into COPRO III, which in turn inhibits CPOX. The experimental data were thus fitted to a substrate-inhibition model (Figure 1B, black line) (eqn 2):

(2) $$V=\frac{V_{\max}\cdot \left[COPROgenIII\right]}{K_{M}+\left[COPROgenIII\right]\cdot \left(1+([COPROgenIII/K_{\mathrm{IS}}])\right)},$$

where *V*_max_ and *K*_M_ are the kinetic parameters of CPOX, and *K*_IS_ (200 ± 35 μM) is the apparent inhibition constant of COPRO III, which reflects both the oxidation rate under the assay conditions and the affinity of the oxidized species for the enzyme.

### COPRO III inhibits CPOX with high affinity

**Figure 2:**
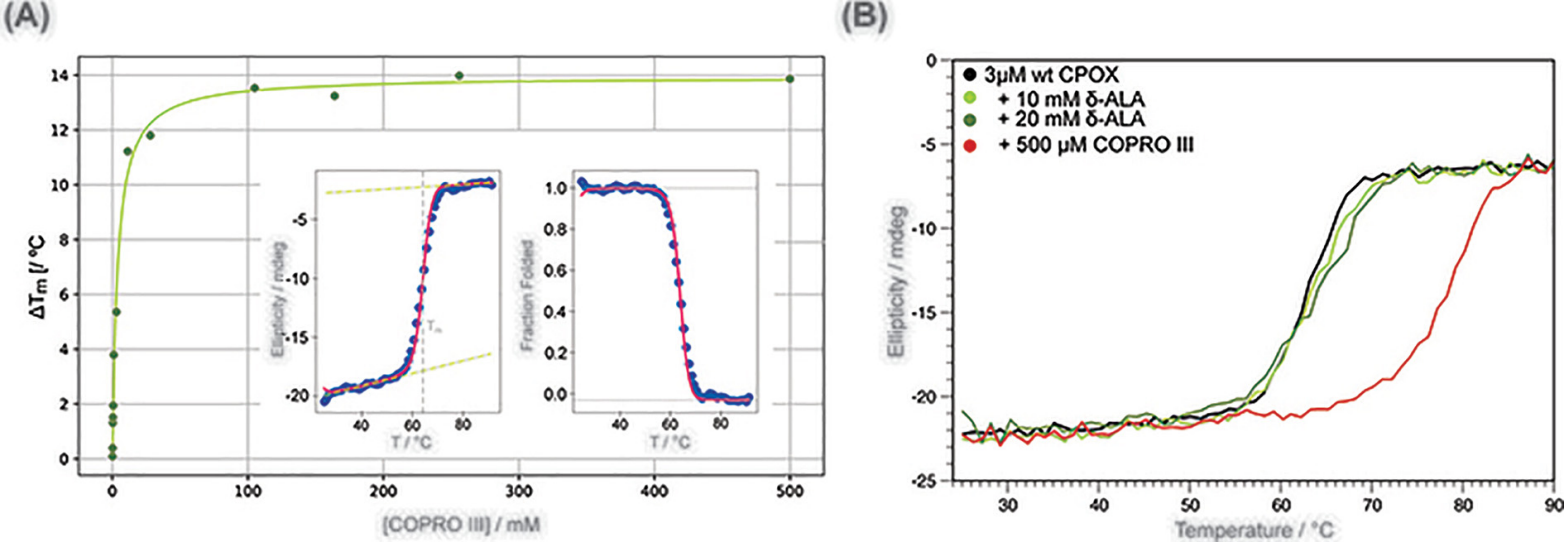
CPOX thermal stability (**A**) Change in the *T*_m_ as a function of the concentration of COPRO III. The green line represents the best fit to the binding model. Inset: representative example for the CD data and the linear extrapolation method to extract the *T*_m_. (**B**) Variation of the *T*_m_ as a function of ligands as indicated in the legend. The solid lines correspond to experimental data.

To investigate the binding of COPRO III to CPOX, we used CD spectroscopy. The thermal melting point (*T*_m_) of apo-CPOX is 62°C (Figure 2A, inset), and it increases by up to 13.7°C upon saturation with COPRO III, reflecting intermolecular association (Figure 2B). Plotting *T*_m_ as a function of titrant concentration produced a sharp sigmoidal curve, which was used to extract the binding constant (Figure 2A). Assuming a single binding site, COPRO III exhibited a dissociation constant (*K*_D_) of 2.1 ± 0.6 μM for its interaction with CPOX.

**Figure 3:**
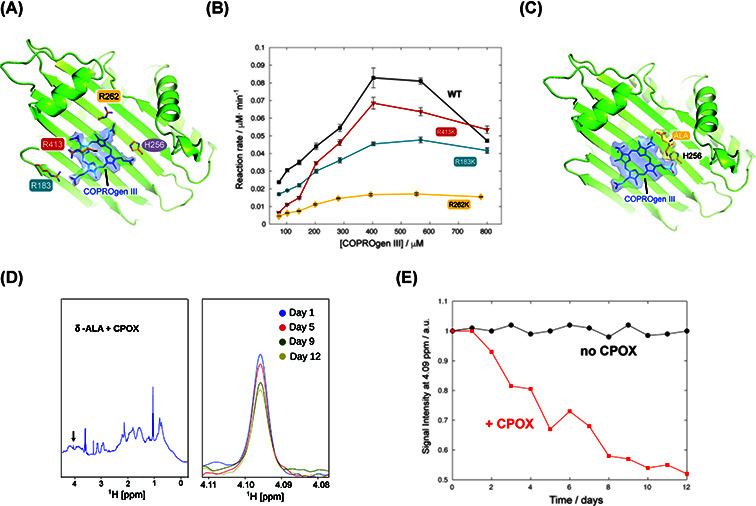
Structural determinants of Coprogen III inhibition of CPOX (**A**) Docking model of human CPOX with the best pose for COPROgen III. Arginines that may produce stabilizing interactions are highlighted and color labeled, as indicated. (**B**) enzyme kinetics experiments for the arginine mutations to lysines, as indicated. (**C**) Docking model of human CPOX with the best pose for COPROgen III and δ-ALA. The catalytic histidine residue is highlighted. (**D**) NMR spectrum showing the time evolution of the combination of CPOX and δ-ALA, as indicated in the legend. The arrow in the left panel shows the position of the δ-ALA signal that was monitored for quantification. (**E**) Stability of δ-ALA (20 μM) at 4°C, in the absence (20 mM phosphate buffer at pH 7.0, 100 mM NaCl, black) or in the same buffer but adding 200 μM of CPOX (red).

Next, we investigated the putative binding location of COPRO III within the enzyme structure. Because COPRO III is structurally analogous to COPROgen III (Figure 1A), it is plausible that the byproduct competes with the substrate for binding to the enzyme. Consistent with this notion, CPOX is known to process related porphyrinogen intermediates, including monovinyl monopropionate porphyrinogen, as alternative substrates [28]. In the absence of a high-resolution enzyme–substrate structure, we generated a docking model of the COPROgen III–CPOX complex (Figure 3A). The docking model positions COPRO III within the active-site cavity in proximity to the catalytic histidine H256 and reveals electrostatic interactions between the porphyrin carboxylate groups and the guanidino side-chain moieties of residues R183, R282, and R413 (Figure 3A). To probe the functional relevance of these interactions, each arginine residue was individually substituted with lysine. Notably, the effects of these mutations on enzymatic activity were not uniform. While the R183K and R413K variants retained substantial catalytic activity with only modest reductions relative to wild type, the R282K mutation located on the same β-strand as the catalytically critical residue H256 led to a pronounced loss of enzymatic activity (Figure 3B).

### δ-ALA inhibits CPOX with low affinity but irreversibly

Indirect evidence for δ-ALA binding to CPOX comes from the crystal structure of the enzyme, which reveals a citrate molecule in the binding site. Since citrate is chemically analogous to δ-ALA, computational docking analysis placed δ-ALA in the same position and orientation as citrate (Figure 3C). We next examined the catalytic efficiency of CPOX in the presence of increasing δ-ALA concentrations under the same experimental conditions (Figure 1B, red circles). At concentrations above 5 mM, δ-ALA reduced CPOX activity. Inspection of Figure 1B indicates that δ-ALA primarily decreases *k*_cat_ while leaving *K*_M_ unchanged, consistent with a non-competitive inhibition mechanism. Indeed, docking suggests that δ-ALA can bind in the active site without preventing substrate (or COPRO III) accommodation but close to the catalytic histidine (Figure 3C).

Accordingly, the data were fitted to a model combining non-competitive inhibition by δ-ALA with substrate inhibition by COPRO III (Figure 1B, red solid lines) (eqn 3):

(3) $$V=\frac{V_{max}\cdot [COPROgenIII]}{\left(K_{M}+[COPROgenIII]\cdot (1+([COPROgenIII/K_{\mathrm{IS}}])))\cdot (1+([\delta -ALA/K_{I}])\right)},$$

where *K*_I_ is the inhibition constant of δ-ALA, determined to be 2.3 ± 0.4 mM.

Under physiological conditions, δ-ALA is unstable and tends to form pyrazines either by self-dimerization or by reacting with other amino acids [29]. To test the possibility of a covalent association between δ-ALA and CPOX, we performed stability studies of δ-ALA in the presence of CPOX at 4°C, a condition chosen to minimize spontaneous degradation. Under the tested conditions, the NMR signal intensity of δ-ALA decayed only in the presence of CPOX (Figure 3D,E). Since no precipitation nor line broadening was observed, the most plausible explanation is a covalent association of δ-ALA with the enzyme. The large molecular weight of the δ-ALA-CPOX complex (∼76 kDa) likely causes the dissapearance of the NMR signal due to slow molecular tumbling. Supporting this conclusion, δ-ALA did not alter the thermal melting point of CPOX at any concentration (Figure 2B), suggesting that it contributes equally to the stability of both folded and unfolded states.

### COPRO III accumulation in a murine HT1 model

**Figure 4:**
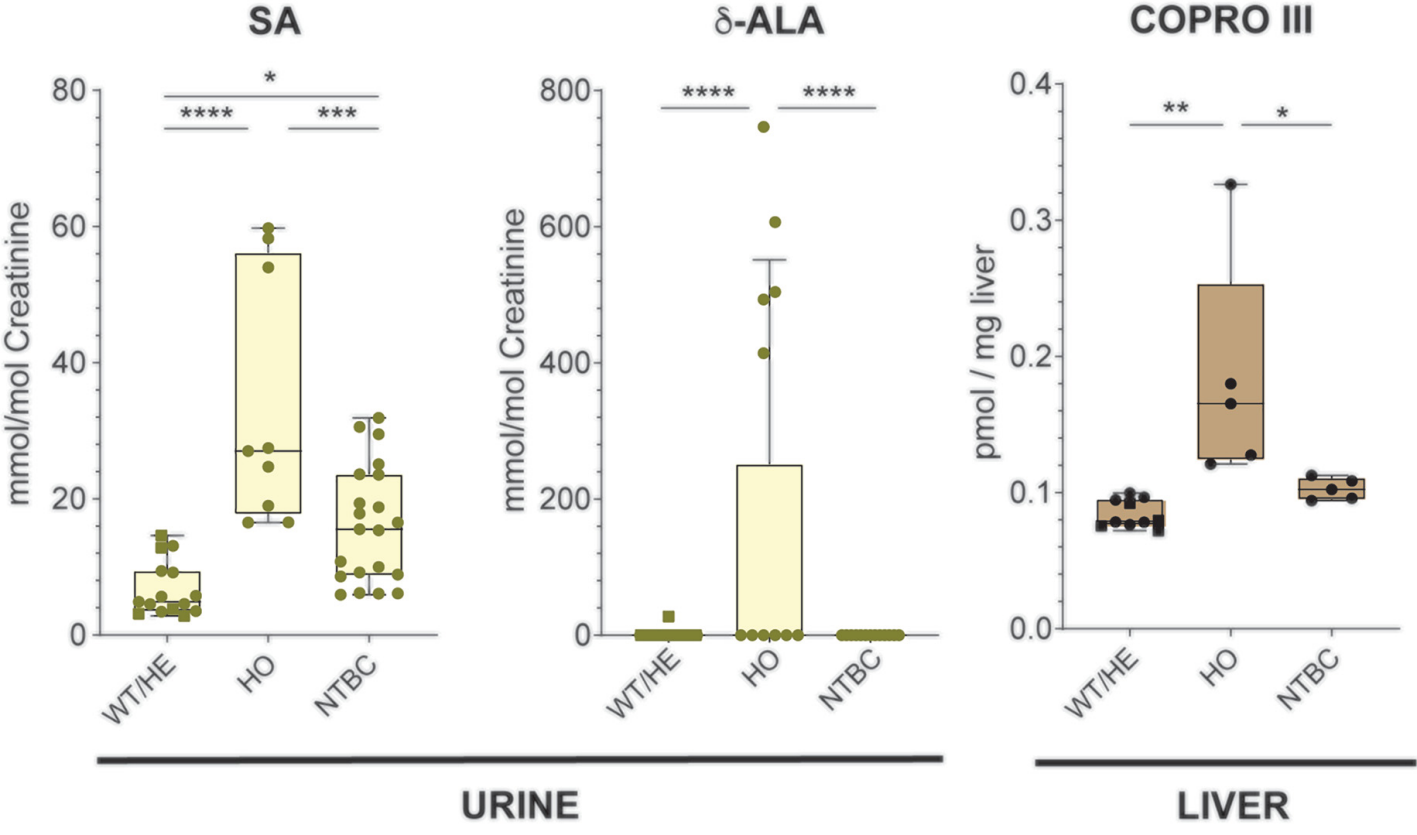
Metabolite accumulation in HT1 mice Concentrations of urine (SA and δ-ALA) and liver tissue (COPRO III) metabolites across groups. Legend for groups: WT/HE: wild-type and heterozygous mice; HO: untreated homozygous mice; NTBC: NTBC-administered HO mice; Legend for *P*-values: * <0.05; ** <0.01; *** <0.001; **** <0.0001.

To assess the potential biological relevance of δ-ALA-mediated CPOX inhibition, we examined a murine model of HT1 carrying a mutation in the fumarylacetoacetate hydrolase (FAH) gene (*fah*^G337S/G337S^). This mutation impairs tyrosine degradation [30], leading to the accumulation of succinylacetoacetate and maleylacetoacetate [31], both of which can decompose chemically to produce succinylacetone (SA). SA is a potent inhibitor of ALAD, ultimately resulting in elevated δ-ALA levels, as confirmed by NMR analysis of urine from the mutant mice (Figure 4).

We next analyzed liver tissue from these mice and observed markedly elevated COPRO III levels compared with control animals (Figure 4). This finding is consistent with δ-ALA-mediated inhibition of CPOX *in vivo* and provides a mechanistic explanation for the porphyria crises reported in HT1 patients [12,32].

Treatment with nitisinone (2-[2-nitro-4-trifluoromethylbenzoyl]-1,3-cyclohexanedione, NTBC) effectively blocks the pathway upstream of FAH by inhibiting 4-hydroxyphenylpyruvate dioxygenase, thereby preventing the accumulation of the intermediates and SA [33]. Consistently, NTBC-treated mice showed neither urinary δ-ALA accumulation nor hepatic COPRO III elevation (Figure 4), underscoring the requirement of high δ-ALA levels for COPRO III accumulation.

### Pb(Ac)_2_ inactivates CPOX

Finally, we examined the effect of Pb^2+^ on CPOX catalytic activity (Figure 1C). Even at low concentrations, the metal markedly reduced enzymatic activity. The Michaelis–Menten profiles still indicated inhibition by the oxidized substrate, suggesting that this mechanism remains operative. Since CPOX lacks canonical metal-binding sites, we hypothesize that Pb^2+^ interacts with molecular oxygen to generate a highly reactive radical species, which in turn inactivates CPOX through oxidative damage to the enzyme.

## Discussion

Rationalizing the accumulation of metabolites in the heme biosynthetic pathway has not only academic but also clinical value, as these accumulated metabolites and by-products degrade into reactive oxygen species that accumulate in the skin and other organs, accounting for much of the pathophysiology of porphyrias and other heme-related diseases. In this context, we investigated the effects of δ-ALA and the by-product COPRO III and Pb^2+^ on the catalytic activity of CPOX.

δ-ALA binds non-competitively to CPOX with low affinity, requiring high concentrations to significantly affect enzymatic activity. However, under pathological conditions such as porphyria, reduced heme availability triggers its own overproduction [34], leading to the accumulation of intermediates at levels sufficient to cause molecular dysfunction. Our data suggest that δ-ALA inhibits CPOX, likely through covalent association, thereby amplifying its inhibitory effect. Given that CPOX is located in the mitochondrial membrane facing the intermembrane space, it has access to δ-ALA from both mitochondrial synthesis and cytosolic back-diffusion. Accordingly, intracellular δ-ALA accumulation in acute hepatic porphyrias, ALAD porphyria, HT1, and in conditions with secondary ALA overproduction such as lead exposure, can be pathogenic *in vivo*, as supported by our analysis of liver tissue from HT1 mice. Biologically, this mechanism is important to understand the effect of δ-ALA and COPRO III accumulation not only in ALAD porphyria but also in other acute hepatic porphyrias (AIP, HCP, and VP) where ALA predominates and in HT1: we hypothesize that δ-ALA buildup leads to CPOX dysfunction, which in turn promotes COPRO III accumulation (favored by the oxidative environment), and this metabolite further inhibits CPOX, creating a synergistic inhibitory loop.

We also found that COPRO III is a potent inhibitor of CPOX, acting competitively with the substrate. Docking analysis indicates that COPRO III binds to a flat surface of the enzyme, where it interacts with stabilizing residues and positions the porphyrin close to the catalytic site. Nonetheless, inspection of the available CPOX structure [20] suggests that local structural rearrangements shall be required for inhibition, particularly involving a helix that partially obstructs product release. This provides a plausible rationale for the inhibitory effect of COPRO III, a substrate analogue.

Finally, we observed that oxidized lead (Pb^2+^) effectively inactivates CPOX, most likely through non-specific redox mechanisms mediated by activated oxygen species. This is consistent with the established ability of lead to inactivate other enzymes of the heme biosynthetic pathway and suggests that porphyrin accumulation in ALAD porphyria and after lead intoxication arises through distinct mechanisms [35].

In clinical lead intoxication, ALAD is the enzyme in the heme pathway that is most susceptible to the inhibitory effects of lead [36] and ALAD inhibition causes marked δ-ALA accumulation, accompanied by increased urinary coproporphyrin III and elevated zinc-protoporphyrin due to impaired iron insertion by ferrochelatase. Thus, lead-induced δ-ALA overproduction may itself contribute to secondary coproporphyrinuria, complementing the direct oxidative inactivation of CPOX observed *in vitro*.

The present study has some limitations. Despite extensive precautions, our experimental conditions favor the reoxidation of COPROgen III into COPRO III, a process that may not occur as readily *in vivo*. Therefore, the relative contributions of δ-ALA versus secondary COPRO III accumulation to the overall phenotype remain uncertain. Moreover, it has been suggested that many mitochondrial proteins involved in heme metabolism form a metabolon [37]. Although CPOX has not been definitively identified as part of such complexes, metabolic compartmentalization could restrict metabolite diffusion, implying that the kinetic parameters reported here should be interpreted qualitatively rather than quantitatively.

In summary, our findings highlight multiple layers of CPOX regulation by endogenous metabolites and exogenous toxins. δ-ALA, COPRO III, and Pb^2+^ each impair CPOX activity through distinct mechanisms (non-competitive, competitive, and oxidative inactivation) and provide a mechanistic framework to explain how metabolic imbalances and environmental exposures contribute to COPRO III accumulation in ALADP and after lead intoxication.

## Supplementary Material

Supplementary Figures S1-S3 and Tables S1-S2

## Data Availability

All data is available upon demand to the corresponding author.

## Abbreviations

δ-ALA: δ-aminolevulinic acid
ALADP: δ-aminolevulinic acid dehydratase deficiency porphyria
CD: circular dichroism
COPRO III: coproporphyrin III
COPROgen III: coproporphyrinogen III
CPOX: coproporphyrinogen oxidase
FAH: fumarylacetoacetate hydrolase
HT1: hereditary tyrosinemia type I
SA: succinylacetone
*T*_m_: thermal melting point
