# Supplementary material for: Aminolevulinate inhibition of human coproporphyrinogen oxidase clarifies coproporphyrin III accumulation in porphyrias
Source: Biosci Rep. 2026 Feb 24;46(3):BSR20254015. doi: 10.1042/BSR20254015 (PMC13071380; doi:10.1042/BSR20254015)
Supplement: Supplementary Figures S1-S3 and Tables S1-S2 [file BSR-2025-4015_supp.pdf]

Supplementary material for:

Aminolevulinate Inhibition of Human Coproporphyrinogen Oxidase clarifies Coproporphyrin III Accumulation in Porphyrias

Andreas Schedlbauer[1], Sarah Kratzwald[2], Margarita Gómez-Galán[1], Jon Gil-Martínez[1,2], Itxaso San Juan[1], Tania Pereira-Ortuzar[1], Fernando Lopitz-Otsoa[1], David Fernandez-Ramos[1], José M Mato[1,3] and Oscar Millet[1,2,3,*]

[1]Precision Medicine and Metabolism Laboratory, CIC bioGUNE, Basque Research and Technology Alliance, Parque Tecnológico de Bizkaia, Ed. 800. 48160, Derio, Spain.

[2]ATLAS Molecular Pharma, Parque Tecnológico de Bizkaia, Ed. 800. 48160, Derio, Spain.

[3]CIBERehd, Spain.

*Corresponding Author:
Oscar Millet
Precision Medicine and Metabolism Laboratory
CIC bioGUNE
+34 956 572 504
omillet@cicbiogune.es

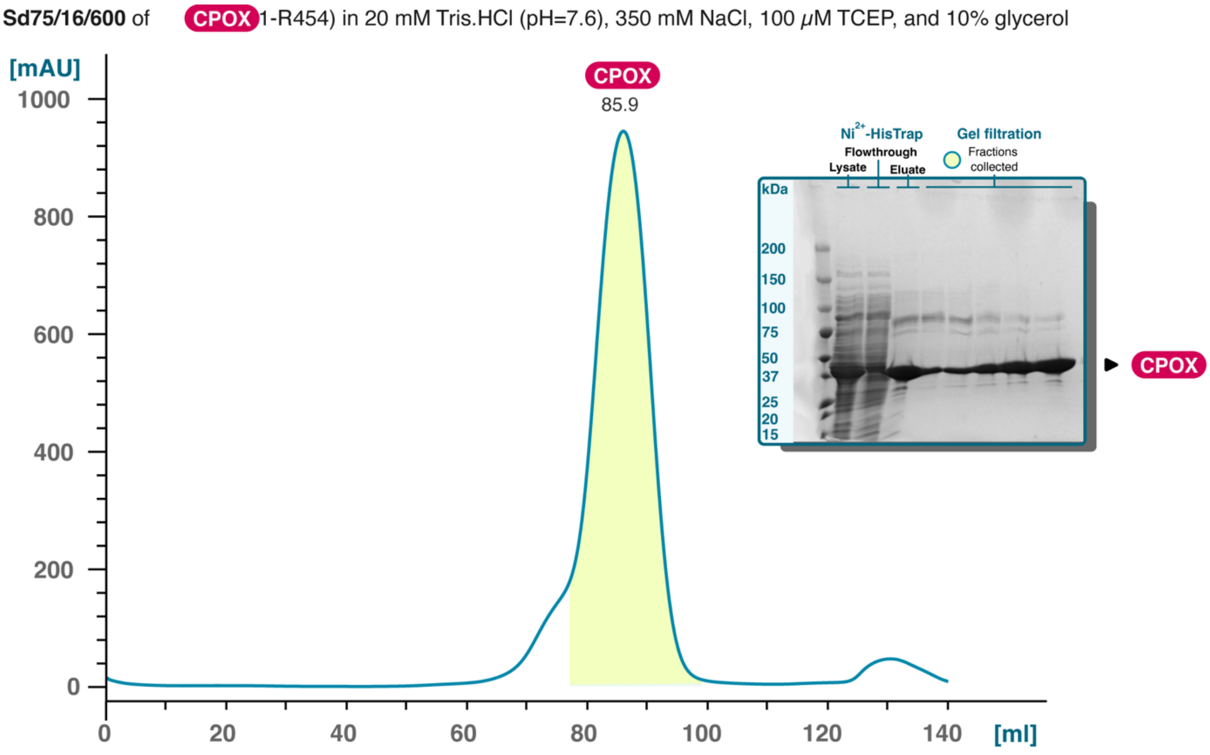

Figure S1. Elution of CPOX in a size exclusion chromatography. The green shadowed area was selected. Inste: SDS analysis of CPOX.

Table S1. HPLC chromatographic and detection conditions for analysis of coproporphyrin III and protoporphyrin IX.

Parameter	Settings
**Injection volume**	50 µl sample volume
**Separation column**	LiChrospher 100 RP-18 (5 mm)
**Solvent A**	90% methanol (v/v) in 1 M ammonium acetate, pH 5.16
**Solvent B**	methanol LiChrosolve (Merck)
**Flow rate**	1.0 ml/min
**Pressure**	Variable
**Temperature**	29°C
**Run time**	30 min
**Fluorimetric detection**	excitation 405 nm; emission 620 nm
**UV detection**	396 nm for coproporphyrin III and 400 nm for protoporphyrin IX

Table 2. Settings applied for Circular Dichroism analysis.

Parameter	Settings
**Measure Range**	200 - 280 nm
**Data pitch**	0.2 nm
**Digital integration time**	4-8 sec
**Bandwidth**	1.00-2.00 nm
**Monitor Wavelength**	220 nm
**Scanning Speed**	50 nm/min
**Temperature ramp**	25°C-90°C
**CD Detector**	PMT
**Cell Length**	1 mm
**Rate of heating**	1.5°C/min

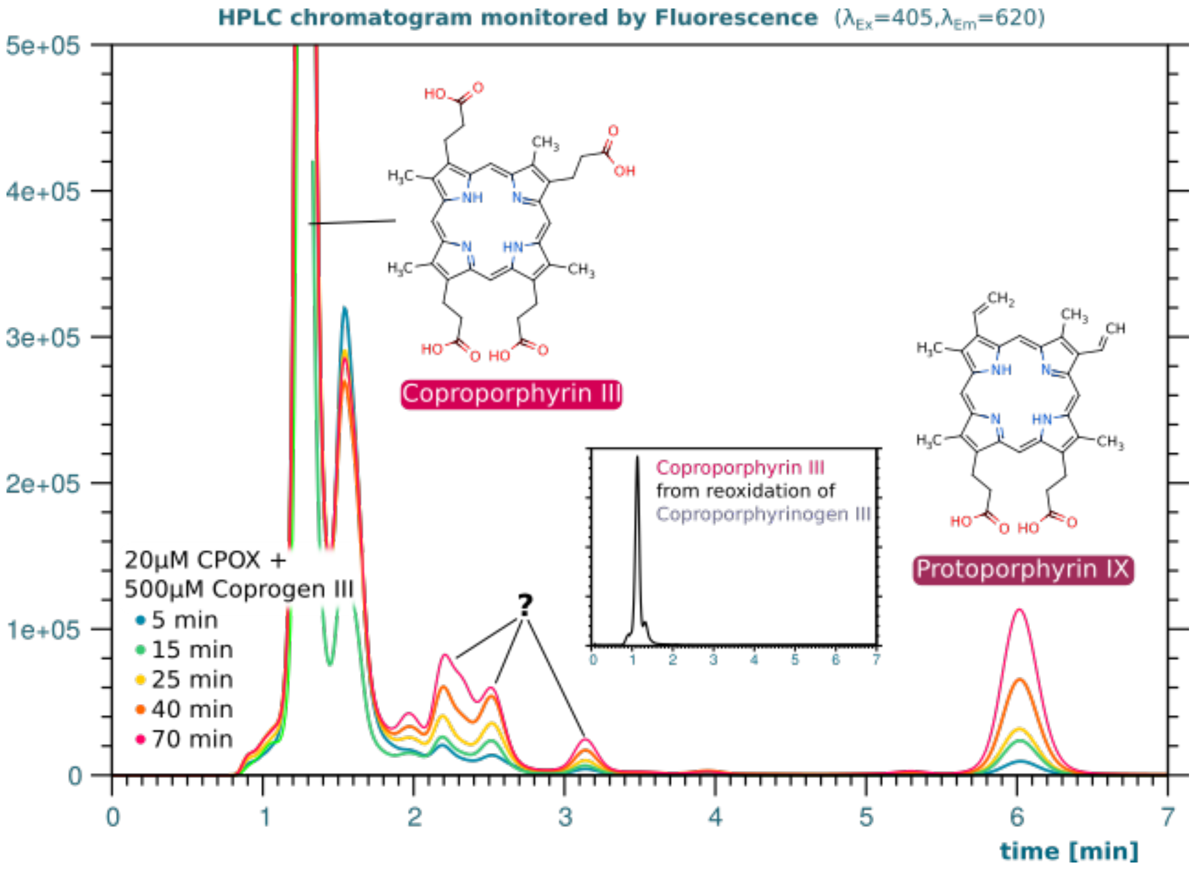

Figure S2. Representative HPLC chromatograms of the CPOX substrate and product. After a reaction time of about one hour at 37°C a peak high for detected product protoporphyrin IX of about 19% relative to the peak amplitude of Coproporphyrin III was observed. Times when the reactions were terminated with 20 % (w/v) trichloroacetic acid in 50 % (v/v) dimethylsulfoxide are indicated. There were some more educt peaks close to Coproporphyrin III showing up that were absent in the chromatogram of the re-oxidized Coproporphyrinogen III substrate (see insert).

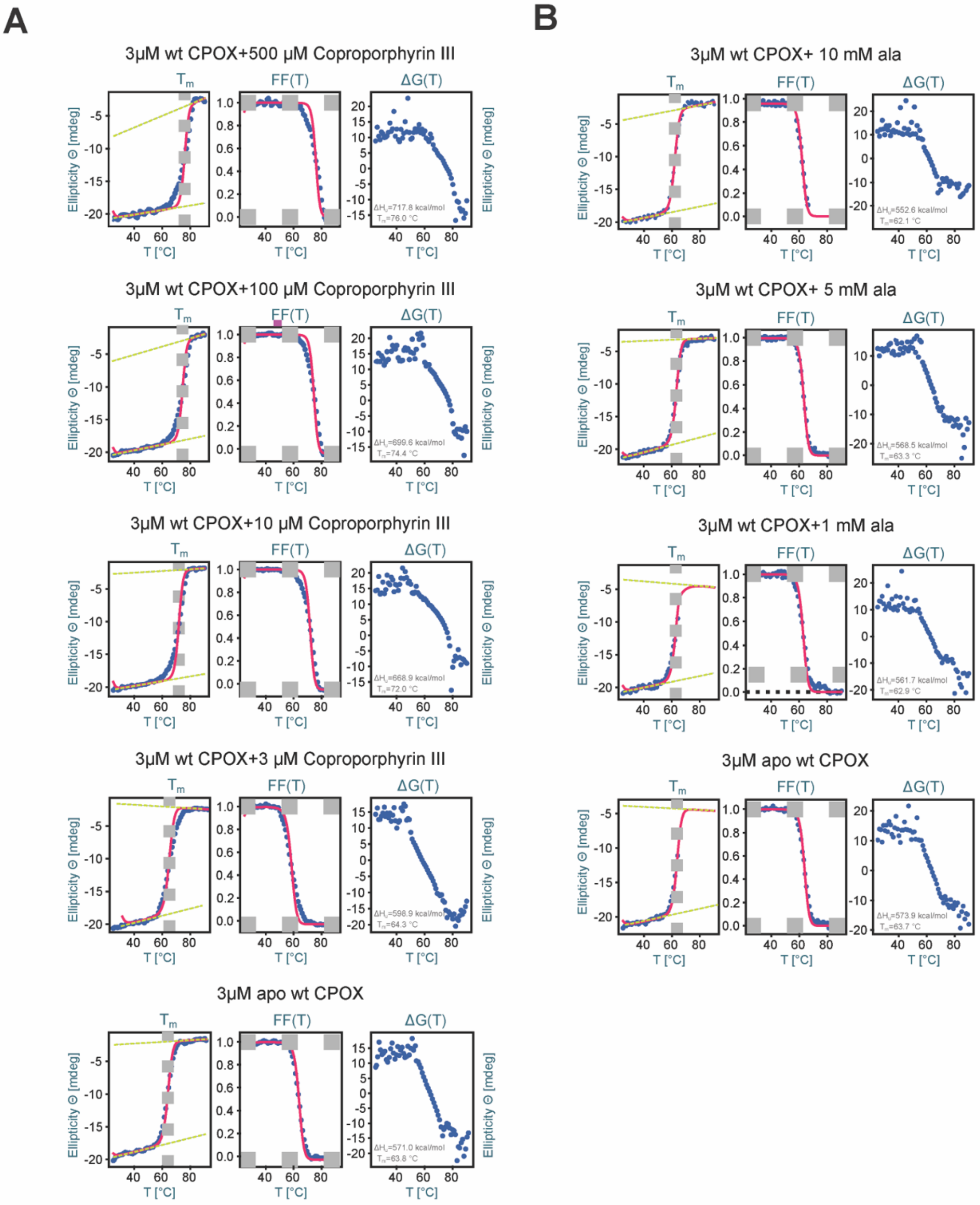

Figure S3. Thermal melt assays. Thermal melt denaturation curves for apo CPOX and the inhibition of COPRO III (A) and δ-ALA (B). Experimental data correspond to filled blue circles, while fitted models (linear extrapolation method). are depicted by pink lines. The green solid lines represent the baseline models. The plots also show the estimation of the free energy as a function of temperature.
